# Dynamic assessment of measurable residual disease in favorable-risk acute myeloid leukemia in first remission, treatment, and outcomes

**DOI:** 10.1038/s41408-021-00591-4

**Published:** 2021-12-06

**Authors:** Sijian Yu, Tong Lin, Danian Nie, Yu Zhang, Zhiqiang Sun, Qing Zhang, Caixia Wang, Mujun Xiong, Zhiping Fan, Fen Huang, Na Xu, Hui Liu, Guopan Yu, Hongyu Zhang, Pengcheng Shi, Jun Xu, Li Xuan, Ziwen Guo, Meiqing Wu, Lijie Han, Yiying Xiong, Jing Sun, Yu Wang, Qifa Liu

**Affiliations:** 1 Department of Hematology, Nanfang Hospital, Southern Medical University, Guangzhou, China; 2 Department of Hematology, Sun Yat-Sen Memorial Hospital, Guangzhou, China; 3 Department of Hematology, Shenzhen Hospital of Southern Medical University, Shenzhen, China; 4 Department of Hematology, Guangdong Second Provincial General Hospital, Guangzhou, China; 5 Department of Hematology, Guangzhou First People’s Hospital, Guangzhou, China; 6 Department of Hematology, The First People’s Hospital of Chenzhou, Chenzhou, China; 7 Department of Hematology, Shenzhen Hospital of Peking University, Shenzhen, China; 8 Department of Hematology, Zhongshan People’s Hospital, Zhongshan, China; 9 Department of Hematology, The First Affiliated Hospital of Guangxi Medical University, Nanning, China; 10 Department of Hematology, The First Affiliated Hospital of Zhengzhou University, Zhengzhou, China; 11 Department of Hematology, The First Affiliated Hospital of Chongqing Medical University, Chongqing, China; 12 Peking University People’s Hospital, Peking University Institute of Hematology, Beijing, China

**Keywords:** Acute myeloid leukaemia, Acute myeloid leukaemia

## Abstract

We aimed to investigate outcomes of different post-remission treatment (PRT) choices based on dynamic measurable residual disease (MRD) by multiparameter flow cytometry in favorable-risk AML (FR-AML). Four hundred and three younger patients with FR-AML in first complete remission (CR1) were enrolled in this registry-based cohort study, including 173 who received chemotherapy (CMT), 92 autologous stem cell transplantation (auto-SCT), and 138 allogeneic SCT (allo-SCT). The primary endpoint was the 5-year overall survival (OS). Subgroup analyses were performed based on dynamic MRD after the 1st, 2nd, and 3rd courses of chemotherapy. In subgroups of patients with negative MRD after 1 or 2 course of chemotherapy, comparable OS was observed among the CMT, auto-SCT, and allo-SCT groups (*p* = 0.340; *p* = 0.627, respectively). But CMT and auto-SCT had better graft-versus-host-disease-free, relapse-free survival (GRFS) than allo-SCT in both subgroups. For patients with negative MRD after three courses of chemotherapy, allo-SCT had better disease-free-survival than CMT (*p* = 0.009). However, OS was comparable among the three groups (*p* = 0.656). For patients with persistently positive MRD after 3 courses of chemotherapy or recurrent MRD, allo-SCT had better OS than CMT and auto-SCT (*p* = 0.011; *p* = 0.029, respectively). Dynamic MRD might improve therapy stratification and optimize PRT selection for FR-AML in CR1.

## Introduction

The management of acute myeloid leukemia (AML) usually includes induction therapy followed by post-remission treatment (PRT) [1, 2]. Approximately 70% of complete morphologic remission (CR) rates were reported in younger AML patients when receiving standard “3 + 7” induction [3–5]. After CR, PRT would be necessary to prevent relapse, which usually consists of several consolidation chemotherapies with or without stem cell transplantation (SCT). Currently, the decision on PRT mainly depends on the risk stratification using cytogenetics and molecular markers [1]. According to genetics-based risk stratification, patients with AML were classified into favorable-risk (FR), intermediate-risk, and poor-risk groups [1]. For FR-AML, intensive chemotherapy is usually recommended as PRT in first CR (CR1) [1]. However, controversy remains regarding the choice of PRT [6–12]. For example, Schlenk et al reported that allogeneic SCT (allo-SCT) and autologous SCT (auto-SCT) both had superior survival than chemotherapy in FR-AML with double mutant CEBPA [6]. Whereas Ahn et al reported that chemotherapy had similar survival to allo-SCT in this subtype [11]. Therefore, there is still a need for additional parameters that can further stratify patients with FR-AML to identify who would benefit from chemotherapy or SCT.

An increasing number of evidence indicate that the presence of measurable residual disease (MRD) identifies a subgroup of patients that is at high risk of relapse and with poor survival [13–18]. Therefore, MRD has been used as an important factor for guiding the choice of PRT [19–22]. But when is the best timepoint making decisions on PRT is still inconclusive [19, 20, 23, 24]. Recently, our multicenter, large-sample study demonstrated that treatment based on dynamic MRD by multiparameter flow cytometry (MFC) was associated with improved outcomes for intermediate-risk AML [25]. Whether dynamic MRD would play an analogous role in FR-AML is unclear. In this study, we retrospectively analyzed a large dataset to explore the clinical significance of dynamic MRD on the choice of PRT for younger FR-AML patients in CR1.

## Patients and methods

### Patients

From January 1, 2012 to December 30, 2017, a total of 642 consecutive patients with newly diagnosed de novo FR-AML in the South China Hematology Alliance database were screened, and 403 patients in CR1 were included in analyses. The definition of FR-AML was based on NCCN criteria [1], which included NPM1mutation, RUNX1-RUNX1T1, CBFB-MYH11, and biallelic mutation of CEBPA. Based on PRT, patients were categorized into three groups: chemotherapy (CMT), auto-SCT, and allo-SCT. Patients who received at least two cycles of consolidation chemotherapy and were not scheduled for upfront SCT were included in the CMT group. Patients who relapsed following CMT and received subsequent SCT were also included in the CMT group. The criteria of enrollment included the following: (1) aged 14–60 years; (2) FR-AML; (3) CR1. Exclusion criteria included the following: (1) acute promyelocytic leukemia; (2) failed to achieve CR after two courses of induction chemotherapy; (3) less than two cycles of consolidation in the CMT group; (4) lack of MRD parameters. Patients with NPM1/FLT3-ITD mutation were also excluded (*n* = 85) due to the small proportion of patients who had results of FLT3-ITD allelic ratio. Moreover, some patients with FLT3-ITD mutation received Sorafenib treatment with little consensus, which may cause unavoidable bias. The endpoint of last follow-up was May 31, 2021. The study complied with the new Helsinki declaration and was reviewed by the ethics committee of Nanfang Hospital, Sun Yat-Sen Memorial Hospital, Shenzhen Hospital of Southern Medical University, Guangdong Second Provincial General Hospital, Guangzhou First People’s Hospital, The First People’s Hospital of Chenzhou, The First Affiliated Hospital of Zhengzhou University, Shenzhen Hospital of Peking University, The First Affiliated Hospital of Guangxi Medical University, Zhongshan People’s Hospital, and The First Affiliated Hospital of Chongqing Medical University, and all patients signed the informed consent.

### Genetic assessment and MRD monitoring

Cytogenetic and molecular analyses were routinely performed at initial diagnosis [25]. After CR, MRD in bone marrow was assessed by eight-color multiparameter flow cytometry (MFC) after induction and each course of PRT and then at two-month intervals within the 1st year, three-month intervals within the 2nd year, four-month intervals within the 3rd year, and half-year intervals from the 4th to 5th year post-treatment [26, 27]. A threshold of 0.1% was employed by MFC-MRD to distinguish MRD-positivity (MRD+) from MRD-negativity (MRD−). MRD by real-time quantitative PCR (RT-qPCR) was also evaluated in a proportion of patients with RUNX1-RUNX1T1, and CBFB-MYH11 with a threshold of 0.01%.

### Treatment procedures

According to our practical guidelines, patients are generally scheduled for “3 + 7” induction therapy consisting of daunorubicin 60 mg/m2 or idarubicin 10–12 mg/m2 on days 1-3 and cytarabine 200 mg/m2 per day for 7 days. For those who failed to achieve CR after the first induction, a second induction consisting of daunorubicin 60 mg/m^2^ or idarubicin 10 mg/m^2^ per day on days 1–3 and cytarabine 2.0 g/m^2^ twice daily on days 1–3 (“3 + 3” regimen), or the same regimen as the first induction was administered [25]. After CR, usually four courses of cytarabine-based consolidation chemotherapy, three courses of chemotherapy followed by auto-SCT, or two courses followed by allo-SCT were administered based on MRD status and donor availability. Cytarabine-based consolidation included “3 + 3” regimen and intermediate/high-dose cytarabine consisting of cytarabine 2.0–3.0 g/m^2^ twice daily on days 1–3. For patients who relapsed following CMT or auto-SCT, allo-SCT was recommended [28]. In auto-SCT, peripheral blood stem cell grafts were collected after mobilization with EA (etoposide plus intermediate-dose cytarabine) combined with granulocyte colony-stimulating factor. In allo-HSCT, the principle of donor selection and transplant protocol was based on the consensus in China [28–30]. Busulfan-based myeloablative conditioning regimens were used in all patients. The prophylaxis for graft-versus-host-disease (GVHD) was described previously [31, 32].

### Endpoints and definitions

The primary endpoint was 5-year cumulative incidence of overall survival (OS). The second endpoints included cumulative incidence of disease-free survival (DFS), the cumulative incidence of relapse (CIR) and non-relapse mortality (NRM), and GVHD-free, relapse-free survival (GRFS). Relapse was defined by morphologic evidence in the peripheral blood, marrow, or extramedullary sites. DFS was evaluated from CR1 to relapse or death or censored at the last follow-up. OS was evaluated from the start of therapy to death or censored at the last follow-up. CR, GVHD, NRM, and GRFS were defined according to previous literature [25]. MRD1, MRD2, and MRD3 were defined as MRD by MFC after one, two, and three courses, respectively. Those without morphologic CR after cycle one were considered MRD1+ [25].

### Statistical analysis

Variables related to patients, disease, and transplant characteristics among groups were compared using Fisher’s exact test for categorical variables and Mann-Whitney U test for continuous variables. OS, DFS, and GRFS were estimated using the Kaplan-Meier method and compared by the log-rank test. Cumulative incidence curves were used in a competing risk setting with relapse treated as a competing event to calculate NRM probabilities, and NRM as competing risk to calculate relapse. The correlations between MRD at different time points were analyzed by Spearman’s. We analyzed the correlations between MRD1, MRD2, and MRD3, and found that the correlation coefficients were low (MRD1 with MRD2 *r* = 0.473; MRD1 with MRD3 *r* = 0.268; MRD2 with MRD3 *r* = 0.456) between them, so the three serial MRD results were included into the regression model as independent variables. The Cox proportional hazards regression model was used for the analysis of risk factors for time-to-event variables. The Fine and Gray model was used for the analysis of endpoints involving competing risks [33]. All tests were two-sided, with significance set at *p* = 0.05. Stata SE 12.0, R version 3.4.3 and Prism version 9.0.1 were used for all data analysis.

## Results

### Patient demographics and treatment characteristics

**Fig. 1:**
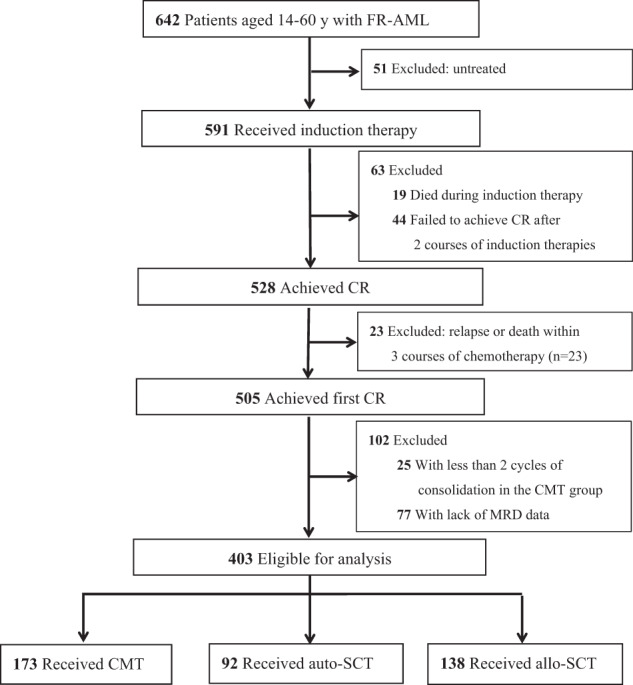
Flow diagram.

A total of 403 patients were enrolled in this study, including 173 in CMT, 92 in auto-SCT, and 138 in allo-SCT groups respectively (Fig. 1 flow diagram). In this study, MRD is a causal variable and ensuring the integrity of MRD data is crucial, so we excluded 77 patients with missing MRD data within 3 courses of chemotherapy. Of the 77 patients, 40 (18.8%) cases were in CMT, 14 (13.2%) in auto-SCT and 23 (14.3%) in allo-SCT groups respectively (*p* = 0.335), indicating that the rates of missing data were comparable among the three groups. In the allo-SCT group, 61 patients were transplanted with matched sibling donors (MSD) and 77 alternative donors (including 64 haploidentical donors (HID), 10 suitably matched unrelated donors (MUD) and 3 umbilical cords blood). The median age was 36 (range: 14–60) years, with 44 (range: 16–60) years in the CMT group, 35 (range: 17–55) years in the auto-SCT group, and 33 (range: 14–60) years in the allo-SCT group. Patients in the CMT group were older than those in auto-SCT and allo-SCT groups (*p* = 0.002, *p* < 0.001, respectively). The proportion of patients needing two cycles to achieve CR in the allo-SCT group was higher than that in the CMT group (*p* = 0.026). More patients were MRD1+, MRD2+, and MRD3+ in the allo-SCT group (*p* = 0.001, *p* = 0.006, *p* < 0.001, respectively). The patient demographics and treatment characteristics for the three groups are presented in Table 1.

**Table 1:** The demographics and treatment characteristics of patients.

*CMT* chemotherapy, *auto-SCT* autologous stem cell transplantation, *allo-SCT* allogeneic stem cell transplantation, *DA* daunorubicin and cytarabine, *IA* idarubicin and cytarabine, *‘3* *+* *3’* daunorubicin or idarubicin 10 mg/m^2^ per day on days 1–3 and cytarabine 2.0 g/m^2^ twice daily on days 1–3, *CR* complete remission, *CR1* first CR, *MRD* measurable residual disease, *MRD1* MRD after one course of chemotherapy, *MRD2* MRD after two courses of chemotherapy, *MRD3* MRD after three courses of chemotherapy.

### Relapse, NRM, and survival

**Fig. 2:**
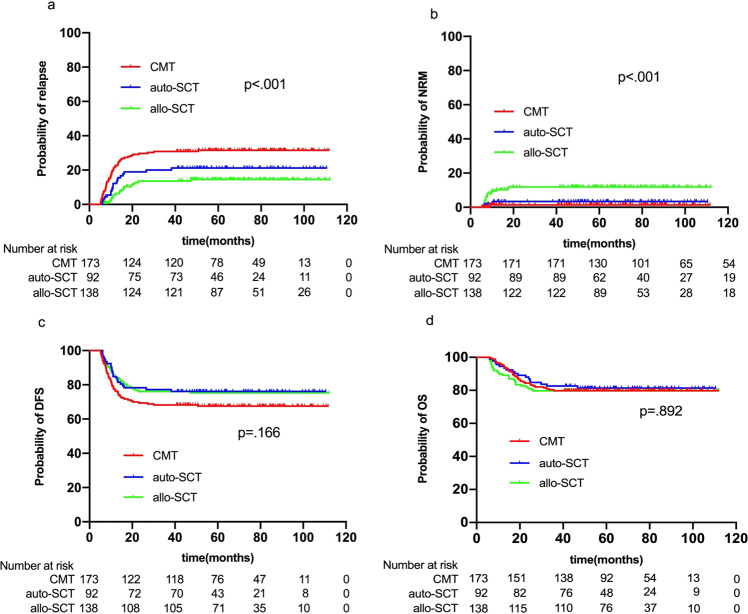
CIR (**a**), NRM (**b**), DFS (**c**), and OS (**d**) for all patients based on different PRT.

The median time from CR1 to relapse was 10.4 (range: 5.2–50.7) months, with 9.7 (range: 5.2–50.7) months in CMT, 11.0 (range: 6.1–38.2) months in auto-SCT, and 14.0 (range: 5.8–47.2) months in allo-SCT groups (*p* = 0.018). The time from CR1 to relapse was much longer in allo-SCT versus CMT (*p* = 0.006), but no statistical significance between allo-SCT and auto-SCT (*p* = 0.181) or between auto-SCT and CMT (*p* = 0.191) was showed. The 5-year CIR was 31.3% (95%CI, 24.5–38.3%) in the CMT group, 20.6% (95%CI, 13.1–29.5%) in the auto-SCT group, and 13.1% (95%CI, 8.1–19.3%) in the allo-SCT group (*p* < 0.001) (Fig. 2a). Multivariate analysis showed that allo-SCT had significantly lower CIR than CMT (HR, 0.176 [95%CI, 0.096–0.324]; *p* < 0.001) and auto-SCT (HR, 0.330 [95%CI, 0.170–0.639]; *p* = 0.001), and auto-SCT had lower CIR than CMT (HR, 0.535 [95%CI, 0.320–0.893]; *p* = 0.017). Two cycles to achieve CR, MRD2+ and MRD3+ were independent risk factors for relapse (Table 2).

**Table 2:** Multivariate analysis for relapse, DFS, and OS.

*WBC* white blood cell, *CR* complete remission, *MRD* measurable residual disease, *MRD1* MRD after one course of chemotherapy, *MRD2* MRD after two courses of chemotherapy, *MRD3* MRD after three courses of chemotherapy, *PRT* post-remission treatment, *CMT* chemotherapy, *auto-SCT* autologous stem cell transplantation, *allo-SCT* allogeneic stem cell transplantation.

The 5-year cumulative incidence of NRM was 1.2% (95%CI, 0.2–3.8%), 3.3% (95%CI, 0.9–8.5%), and 11.6% (95%CI, 6.9–17.6%) in the CMT, auto-SCT and allo-SCT groups, respectively (*p* < 0.001) (Fig. 2b). Allo-SCT had significantly higher NRM than CMT (HR, 10.605 [95%CI, 2.449–45.923]; *p* = 0.002) and auto-SCT (HR, 3.710 [95%CI, 1.080–12.744]; *p* = 0.037), but no difference was found between auto-SCT and CMT (HR, 2.858 [95%CI, 0.478–17.090]; *p* = 0.250).

The 5-year DFS was 67.5% (95%CI, 60.0–74.0%) in the CMT group, 76.1% (95%CI, 66.0–83.6%) in the auto-SCT group, and 75.3% (95%CI, 67.2–81.7%) in the allo-SCT group (*p* = 0.166) (Fig. 2c), which was comparable among the three groups in univariate analysis. However, multivariate analysis showed that allo-SCT (HR, 0.372 [95%CI, 0.234–0.591]; *p* < 0.001) and auto-SCT (HR, 0.595 [95%CI, 0.360–0.984]; *p* = 0.043) had better DFS than CMT. Allo-SCT was associated with comparable DFS as auto-SCT (HR, 0.626 [95%CI, 0.358–1.092]; *p* = 0.099). The 5-year OS was 79.8% (95%CI, 73.0–85.0%), 81.3% (95%CI, 71.7–88.0%), and 79.7% (95%CI, 72.0–85.5%) in the three groups, respectively (*p* = 0.892) (Fig. 2d). PRT was not an independently influential factor in both univariate and multivariate analysis of OS (Fig. 2d; Table 2). Multivariate analysis revealed that higher white blood cell count (≥50 × 10^9^/L), two cycles to achieve CR, MRD2+ and MRD3+ were risk factors for DFS and OS (Table 2).

### Treatment and outcomes after relapse

Ninety-one patients relapsed at last follow-up, including 54 cases in CMT group, 19 in auto-SCT and 18 in allo-SCT groups respectively. Eighty-three patients received reinduction therapy and 66 cases (79.5%) achieved CR2, including 44/51 (CR rate: 86.3%) in the CMT group, 11/16 (68.8%) in the auto-SCT group and 11/16 (68.8%) in the allo-SCT group. A total of 46 patients received allo-SCT (MSD: *n* = 18; HID: *n* = 21; MUD: *n* = 7), including 31 cases in the CMT group, 11 in the auto-SCT group and 4 in the allo-SCT group. Of the 31 patients in the CMT group, 28 achieved CR2 (5-year OS: 60.5% (95%CI, 51.2–69.8%)) and 3 with refractory disease (1 patient survived) before transplantation. Of the 11 patients in the auto-SCT group, 9 achieved CR2 (5-year OS: 44.4% (95%CI, 27.8–61.0%)) and 2 with refractory disease (both dead) before transplantation. Of the 4 patients in the allo-SCT group, all of them achieved CR2 and 2 survived. The overall 5-year OS calculated from the date of relapse were 38.6% (95%CI, 25.7–51.3%) in the CMT group, 26.3% (95%CI, 9.6–46.8%) in the auto-SCT group and 33.3% (95%CI, 13.7–54.5%) in the allo-SCT respectively (*p* = 0.506).

### Dynamic MFC-MRD, PRT selection and outcomes

**Fig. 3:**
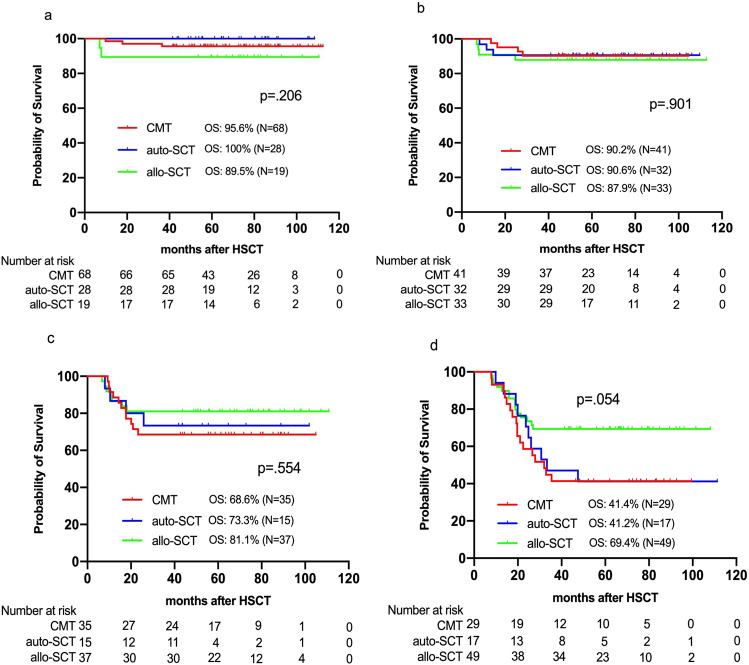
Overall survival in subgroup A (**a**), B (**b**), C (**c**), and D (**d**) based on different PRT.

To explore the association between dynamic MRD by MFC, PRT selection and outcomes for FR-AML, subgroup analyses were performed according to the dynamics of MRD1, MRD2, and MRD3. Patients were classified into four subgroups: (I) subgroup A, MRD- after 1 course of chemotherapy (MRD1−/MRD2−/MRD3−), (II) subgroup B, MRD- after 2 courses of chemotherapy (MRD1+/MRD2−/MRD3−), (III) subgroup C, MRD− after 3 courses of chemotherapy (MRD1+/MRD2+/MRD3−), IV) subgroup D, persistently MRD+ after 3 courses of chemotherapy (MRD1+/MRD2+/MRD3+) or recurrent MRD (from MRD− to MRD+). The univariate analyses of OS in each subgroup were presented in Fig. 3. Furthermore, subgroup analyses were performed after adjustment for various covariates. In subgroup A, comparable CIR (HR, 0.951 [95%CI, 0.426–2.126]; *p* = 0.903), DFS (HR, 1.524 [95%CI, 0.688–3.375]; *p* = 0.300), and OS (HR, 1.808 [95%CI, 0.536–6.097]; *p* = 0.340) were found among the CMT, auto-SCT and allo-SCT groups. Both CMT (HR, 0.282 [95%CI, 0.095–0.840]; *p* = 0.023) and auto-SCT (HR, 0.194 [95%CI, 0.039–0.964]; *p* = 0.045) had better GRFS than allo-SCT. In subgroup B, comparable CIR (HR, 0.526 [95%CI, 0.250–1.106]; *p* = 0.090), DFS (HR, 0.912 [95%CI, 0.481–1.732]; *p* = 0.779), and OS (HR, 1.208 [95%CI, 0.563–2.595]; *p* = 0.627) were also found among the three groups. However, CMT (HR, 0.372 [95%CI, 0.148–0.933]; *p* = 0.035) and auto-SCT (HR, 0.267 [95%CI, 0.087–0.820]; *p* = 0.021) had better GRFS than allo-SCT. In subgroup C, allo-SCT had lower CIR than CMT (HR, 0.115 [95%CI, 0.017–0.773]; *p* = 0.026), resulting in better DFS (HR, 0.249 [95%CI, 0.088–0.703]; *p* = 0.009). However, there was no difference in CIR (HR, 0.305 [95%CI, 0.043–2.162]; *p* = 0.234) and DFS (HR, 0.311 [95%CI, 0.085–1.139]; *p* = 0.078) between auto-SCT and CMT or in CIR (HR, 0.378 [95%CI, 0.071–2.016]; *p* = 0.254) and DFS (HR, 0.802 [95%CI, 0.230–2.793]; *p* = 0.729) between allo-SCT and auto-SCT. There was no difference in OS (HR, 0.872 [95%CI, 0.476–1.597]; *p* = 0.656) and GRFS (HR, 0.846 [95%CI, 0.591–1.211]; *p* = 0.360) among the three groups. In subgroup D, allo-SCT had lower CIR than CMT (HR, 0.120 [95%CI, 0.058–0.249]; *p* < 0.001) and auto-SCT (HR, 0.149 [95%CI, 0.063–0.353]; *p* < 0.001), resulting in advantages in DFS compared with CMT (HR, 0.214 [95%CI, 0.113–0.407]; *p* < 0.001) and auto-SCT (HR, 0.263 [95%CI, 0.117–0.591]; *p* = 0.001), and better OS than CMT (HR, 0.399 [95%CI, 0.197–0.810]; *p* = 0.011) and (HR, 0.380 [95%CI, 0.160–0.906]; *p* = 0.029). Thus, our findings suggested that CMT might be recommended for patients in subgroup A, B, and C due to comparable OS as auto-SCT or allo-SCT while allo-SCT for patients in subgroup D because of better OS than CMT or auto-SCT.

### MFC and RT-qPCR integrated evaluation of MRD3, treatment and outcomes

To further study the association of integrated results of MFC and RT-qPCR MRD, treatment and outcomes, 152 patients with integrated results of MRD3 were analyzed, including 104 cases with RUNX1-RUNX1T1, and 48 cases with CBFB-MYH11. Median MRD3 level by RT-qPCR was 0.048% (range: 0.01–10.96%) in patients with RUNX1-RUNX1T1 and 0.025% (range: 0.01–7.83%) in patients with CBFB-MYH11 (*p* = 0.163). This integrated analysis identified four categories of patients: double negative (MFC−/PCR−), double positive (MFC+/PCR+) and single positive (MFC+/PCR− or MFC−/PCR+). Among subjects who were MFC−/PCR− (*n* = 39), the 5-year OS was 86.2% (95% CI 67.3–94.6) in the CMT group, 100% in the auto-SCT and 100% in the allo-SCT, respectively (*p* = 0.483). Among patients who were MFC−/PCR+ (*n* = 76), the 2-year OS was comparable between the three groups (*p* = 0.684), with 81.2% (95% CI 63.0–91.1) in the CMT group, 77.8% (95% CI 55.1–91.0) in the auto-SCT and 88.5% (95% CI 68.4–96.1) in the allo-SCT, respectively, despite CIR was significantly lower in allo-SCT than CMT (9.1% vs 40.6%, *p* = 0.006). Among two patients who were MFC+/PCR−, one received CMT as consolidation and relapsed and die of relapse; the other one received auto-SCT as PRT and relapsed and die of infection after receiving allo-SCT as salvage treatment. Among patients who were MFC+/PCR+ (*n* = 35), despite lower CIR and better DFS was observed in the allo-SCT compared with that in the CMT group (CIR: 16.7% vs 77.8%, *p* < 0.001; DFS: 75.0% vs 22.2%, *p* = 0.001), the 2-year OS was comparable between the three groups (overall, *p* = 0.160; allo-SCT vs CMT, *p* = 0.072), with 44.4% (95% CI 13.6–71.9) in the CMT group, 50.0% (95% CI 11.1–80.4) in the auto-SCT and 80.0% (95% CI 55.1–92.0) in the allo-SCT, respectively.

## Discussion

In this study, we first attempt to explore optimal PRT choices according to dynamic MFC-MRD for FR-AML. Our findings suggested that: for patients with MRD- within 3 cycles of chemotherapy, CMT might be recommended in CR1; for patients with persistently MRD + after 3 cycles of chemotherapy or recurrent MRD, allo-SCT might be recommended.

For FR-AML in CR1, the guidelines recommend consolidation chemotherapy as first-line treatment [1, 2]. However, some studies reported the beneficial role of auto-SCT or allo-SCT compared with chemotherapy in this subtype [6–9, 19, 34, 35]. Therefore, further therapy stratification after CR would be necessary to identify the optimal PRT choice. MRD has been effectively used for directing PRT [7, 19, 23, 36–38]. Nevertheless, the best timing for treatment choice based on MRD remains inconclusive [19, 23, 24, 34, 39]. For instance, Zhu et al [19] reported that MRD status after the second consolidation might discriminate high-risk relapse patients with t(8;21) AML, for whom allo-SCT could reduce relapse and improve survival compared with chemotherapy. Balsat et al [23] reported that patients with NPM1mutation who didn’t achieve a 4-log reduction in MRD after induction had a higher CIR and benefited from allo-SCT. While Yao et al [24] indicated that MRD after the first consolidation might be the best timing for the choice of PRT. However, these studies all focused on the value of static MRD for treatment options and outcomes. Interestingly, our recent investigation suggested that clinical decisions based on dynamic MRD might be associated with improved therapy stratification and optimized PRT for intermediate-risk AML [25].

In the current study, in order to explore whether dynamic MRD would play an analogous role in FR-AML as in intermediate-risk AML, we retrospectively analyzed 403 younger patients with FR AML from a registered database. Adjusted subgroup analyses according to dynamic MFC-MRD showed that for patients with MRD- within two courses of chemotherapy, comparable CIR, DFS and OS were observed among the three PRT groups. Whereas CMT or auto-SCT was associated with better GRFS as allo-SCT. So CMT might be recommended for this subset due to better GRFS than allo-SCT and comparable OS as auto-SCT or allo-SCT. For patients with MRD- after 3 courses of chemotherapy, allo-SCT had higher DFS than CMT owing to significantly lower CIR in the former, indicating that allo-SCT exert a stronger anti-leukemia effect than CMT for this subset. However, OS and GRFS were comparable among the three groups. For patients in this subset, the better DFS in allo-SCT didn’t translate into advantageous OS, which was mainly attributed to allo-SCT as salvage treatment after relapse, consistent with literature reports [6, 11]. So we might recommend CMT for patients in CR1 and salvage chemotherapy followed by allo-SCT for relapsed patients. For patients with persistently MRD + after 3 cycles of chemotherapy or recurrent MRD, significantly lower CIR, higher LFS and OS were achieved in allo-SCT than CMT or auto-SCT, so allo-SCT might be recommended for this subset.

Both MFC and RT-qPCR are useful techniques during the MRD monitoring of AML [19, 36, 40–44]. Currently, the two main methods of evaluating MRD in AML include MFC and RT-qPCR [19, 36, 40–44]. Because RT-qPCR and MFC identify MRD in fundamentally distinct manners (genomic versus phenotypic aberrations), these methods may be complementary in the assessment of MRD [41, 42, 45]. For instance, Ouyang et al [42] suggested that, while qRT-PCR level between 0.1% to 1% and 1% to 10% failed to predict relapse for patients with core-binding factor AML, MFC provided prognostic value for relapse. However, the therapeutic implications of integrated MRD remain less clear [45]. In this study of favorable-risk AML, we demonstrated that dynamic MFC-MRD may identify a subgroup of patients with high-risk of relapse who may benefit from allo-SCT. However, we failed to investigate the association of dynamic MRD by RT-qPCR or combination of the two techniques, treatment, and outcomes due to the limited number of patients who had complete RT-qPCR MRD data at each of the three-time points. Instead, exploratory analyses were performed based on MRD3, which was a key variable affecting outcomes in this study. Our results suggested that CMT may achieve comparable survival rates with allo-SCT for patients who were MFC-/PCR-, allo-SCT may have favorable survival rates than CMT for patients who were MFC+/PCR+, in accordance with our results by MFC method alone. For patients who were MFC-/PCR+, allo-SCT had lower CIR than CMT, but similar OS was observed between the two. The results suggest that for this subgroup of patients, allo-SCT might not be essential at status of CR1. Two patients who were MFC+/PCR− received treatment of CMT/auto-SCT and both relapsed and died. Our results seem to imply that MFC alone may provide useful clinical information for decision-making. However, the results need to be explained with caution due to several issues, such as the small number of patients in subgroups, potential bias of selection of patients who had qPCR-MRD results and undefined optimal thresholds at different time points for different molecular makers.

Although auto-SCT was a beneficial factor in multivariate analysis of DFS when taking CMT as a reference in the whole cohort, OS was comparable between the two in both univariate and multivariate analyses. In subgroup analyses, only in patients who turned MRD- after 3 courses of chemotherapy, auto-SCT showed a tendency of better DFS than CMT. Whether this subset of patients might benefit from auto-SCT needs to be further investigated because of the relatively small population in subgroup analysis.

### Limitations

This study has several limitations. Firstly, the bias of the retrospective nature was inevitable. For instance, there was an imbalance in patients’ age between CMT and SCT groups. But we tried to address this issue by multivariate analysis. Secondly, in some subgroups, results should be explained with caution due to small numbers. Thirdly, MRD was analyzed and interpreted at each respective institute, which was suggested to be performed at a central institute with plenty of experience [46]. To address these issues and further validate our findings, we have conducted a prospective, multicenter trial on dynamic MRD-directed therapies for AML (NCT 02870777).

## Conclusions

Our results suggest that dynamic MFC-MRD might improve therapy stratification and optimize PRT for FR-AML. CMT might be preferable for patients with MRD− within three cycles of chemotherapy in CR1 and allo-SCT as salvage for patients after relapse; allo-SCT might be recommended for patients with persistently MRD+ after three cycles of chemotherapy and recurrent MRD in CR1.

## Supplementary information

aj-checklist

## Data Availability

All data generated or analyzed during this study are included in this published article.
